# A Randomized Controlled Trial to Evaluate the Benefits of a Multimedia Educational Program for First-Time Hearing Aid Users

**DOI:** 10.1097/AUD.0000000000000237

**Published:** 2016-02-25

**Authors:** Melanie Ferguson, Marian Brandreth, William Brassington, Paul Leighton, Heather Wharrad

**Affiliations:** 1 NIHR Nottingham Hearing Biomedical Research Unit, Otology and Hearing Group, Division of Clinical Neuroscience, School of Medicine, University of Nottingham, Nottingham, United Kingdom; 2 Nottingham University Hospitals NHS Trust, Nottingham, United Kingdom; 3 School of Medicine, and 4 School of Health Sciences, University of Nottingham, Nottingham, United Kingdom.

**Keywords:** Auditory rehabilitation, Education, E-learning, Hearing aid benefit, Hearing loss, Knowledge, Reusable learning objects, Telehealth, Teleaudiology

## Abstract

**Objectives:**

The aims of this study were to (1) develop a series of short interactive videos (or reusable learning objects [RLOs]) covering a broad range of practical and psychosocial issues relevant to the auditory rehabilitation for first-time hearing aid users; (2) establish the accessibility, take-up, acceptability and adherence of the RLOs; and (3) assess the benefits and cost-effectiveness of the RLOs.

**Design:**

The study was a single-center, prospective, randomized controlled trial with two arms. The intervention group (RLO+, n = 103) received the RLOs plus standard clinical service including hearing aid(s) and counseling, and the waitlist control group (RLO−, n = 100) received standard clinical service only. The effectiveness of the RLOs was assessed 6-weeks posthearing aid fitting. Seven RLOs (total duration 1 hr) were developed using a participatory, community of practice approach involving hearing aid users and audiologists. RLOs included video clips, illustrations, animations, photos, sounds and testimonials, and all were subtitled. RLOs were delivered through DVD for TV (50.6%) and PC (15.2%), or via the internet (32.9%).

**Results:**

RLO take-up was 78%. Adherence overall was at least 67%, and 97% in those who attended the 6-week follow-up. Half the participants watched the RLOs two or more times, suggesting self-management of their hearing loss, hearing aids, and communication. The RLOs were rated as highly useful and the majority of participants agreed the RLOs were enjoyable, improved their confidence and were preferable to written information. Postfitting, there was no significant between-group difference in the primary outcome measure, overall hearing aid use. However, there was significantly greater hearing aid use in the RLO+ group for suboptimal users. Furthermore, the RLO+ group had significantly better knowledge of practical and psychosocial issues, and significantly better practical hearing aid skills than the RLO− group.

**Conclusions:**

The RLOs were shown to be beneficial to first-time hearing aid users across a range of quantitative and qualitative measures. This study provides evidence to suggest that the RLOs may provide valuable learning and educational support for first-time hearing aid users and could be used to supplement clinical rehabilitation practice.

## Abstract 2

A series of seven multimedia interactive videos (or reusable learning objects [RLOs]) covering a range of practical and psychosocial information were developed for first-time hearing aid users. A prospective randomized controlled trial of 203 patients showed that the intervention group that received the RLOs had better knowledge of hearing-related issues, better practical hearing aid skills, and greater hearing aid use in suboptimal hearing aid users, compared with the control group at 6 weeks post-fitting. RLOs were rated as highly useful, and user feedback on the RLOs was very positive. Take-up was high and adherence was at least 67%. RLOs provide valuable educational support.

Supplemental Digital Content is available in the text.

## INTRODUCTION

Hearing loss results in speech perception and communication difficulties, which in adults can lead to social withdrawal, depression, employment problems, an increased risk of dementia, and reduced quality of life (Davis et al. 2007; Lin et al. 2011). Provision of hearing aids is the main form of clinical intervention for adults with hearing loss (Kochkin 2009). Other components of aural rehabilitation include instruction on the use of hearing aids and communication strategies, counseling to enhance participation in everyday life, and strategies to enhance speech perception, such as auditory training (Boothroyd 2007).

Despite evidence that hearing aids are effective in providing hearing-specific benefits and improved quality of life (Chisolm et al. 2007; Davis et al. 2007), a significant proportion of hearing aid users, estimated between 4.5 and 24%, do not wear them (McCormack & Fortnum 2013), and others wear them only some of the time (Whitmer et al. 2014). Reasons for this are varied. These include comfort and maintenance of hearing aids, psychosocial and situational influences, device factors, and attitude of healthcare professionals (McCormack & Fortnum 2013). Furthermore, expectations of first-time hearing aid users are often set too high, leading to unrealistic expectations (Wong et al. 2003; Meyer et al. 2014). It is not surprising then that around half (51%) of first-time hearing aid users have difficulties using their hearing aids (Action on Hearing Loss 2011).

Provision of high-quality information by audiologists to hearing aid users, particularly those using them for the first time, may help to address many of the above issues. Typically, much of the information offered in a clinical setting is delivered verbally, with the result that many patients forget the information given to them. Kessels (2003) reported that between 40 and 80% of information was forgotten after the clinic appointment. For hearing aid users, Reese and Hnath-Chisolm (2005) showed that 25% of information delivered at the hearing aid fitting appointment was forgotten 1 month later when assessed using a multiple-choice method. However, El-Molla et al. (2012), using a free recall method, reported that half of the information given was forgotten 6 weeks later, with poorer retention of psychosocial issues (35%) compared with practical issues (65%). These study findings are reflected in a typical comment from a first-time hearing aid user, “you get a lot of information…by the time you get home you’ve forgotten most of it” (Action on Hearing Loss 2011, p. 23).

As follow-up appointments are not routine in the UK National Health Service (Lowe 2015), the importance of high-quality information to reinforce that given at the fitting appointment is important. Although delivery of high-quality written information is recognized and recommended as good clinical practice in the UK (NHS Scotland 2009; Action on Hearing Loss 2011), this is not always done in clinic. A survey of 107 UK audiology services showed that fewer than 40% of services offered written information other than the standard provision of hearing aid manufacturers’ hearing aid user guide (Action on Hearing Loss 2011). However, hearing aid manufacturers’ user guides are not optimal in terms of content, design, and readability (Brooke et al. 2012; Caposecco et al. 2014). It is also important to recognize that one-way delivery of information by the audiologist to the hearing aid user is not the same as educating the patient and increasing their knowledge base (Boothroyd 2007). Constructivist learning theory suggests that learners construct an internal representation by taking an interactive role in learning, and that higher interactivity with learning material promotes better learning (Zhang et al. 2006). Therefore for development of a good knowledge base and subsequent learning to occur, hearing aid users need to not only receive good quality information but also understand and actively engage with the information for it to transfer, and be relevant and useful in their everyday lives.

Knowledge of hearing-related matters in the general public is generally poor (Greengross 2014), and even in existing hearing aid users, knowledge of hearing aids and how to use them is highly variable, ranging from poor to excellent (Desjardins & Doherty 2009). For example, between 60 and 80% of first-time hearing aid users did not know how to use the telephone with their hearing aids and needed further instruction (Vuorialho et al. 2006; Goggins & Day 2009). To address this, a number of educational programs for hearing aid users have been developed. Communication programs, delivered either in group or individual settings, have been shown to provide benefits in terms of reduction of self-reported hearing and communication difficulties (Beynon et al. 1997; Chisolm et al. 2004; Hickson et al. 2007). For some programs, although not all (e.g., Active Communication Education [ACE]), this requires input from the audiologist. This can be costly, both in terms of time and finances, which may be problematic when healthcare resources are limited.

Remote delivery of a more cost-effective individual home-communication program delivered by videotapes showed improvement in the use of communication strategies and improved quality of life and satisfaction at a 6-month follow-up (Kramer et al. 2005). Similarly, a randomized controlled trial (RCT) of a 5-week written educational program that covered the basics of hearing, the audiogram, and information on hearing aids, supplemented by weekly telephone calls, showed a reduction in hearing handicap and reported anxiety in the intervention group in existing hearing aid users (Lundberg et al. 2011). These materials were further developed by Thorén et al. (2011) for internet delivery with email feedback and advice from an audiologist. An RCT of these internet-delivered materials showed no improvements in existing hearing aid users on the Hearing Handicap Inventory for the Elderly (HHIE) in both intervention and control groups; however, there was a reduction in depression symptoms both immediately and at the 6-month follow-up. Recently, these materials were expanded with additional information on hearing and cognition, and components from the ACE (Hickson et al. 2007), as well as online interaction with peers and an audiologist (Thorén et al. 2014). A subsequent RCT showed improvements in the HHIE and two items of the International Outcome Inventory for Hearing Aids (IOI-HA) in the intervention group immediately and at 6-month follow-up. Thus, educational programs appear to be beneficial to hearing aid users.

In other fields, educational and psychological research provides evidence that visual approaches can enhance learning and motivation by providing concrete depictive representations of subjects to be learned (Zhang 1997; Ainsworth & Loizou 2003). In the present study, we investigated the use of reusable learning objects (RLOs). These are “chunks” of interactive multimedia learning, containing highly visual components that include animations, cartoon, and video clips to illustrate concepts and processes (Windle & Wharrad 2010). The RLO concept is based on three components (1) visual illustration of concepts, (2) activity and engagement with content, and (3) self- assessment. Two important principles are that RLOs are based on specific learning goals, and the theoretical framework underpinning the pedagogical design (Koper 2003) ensures that the multimedia environment enables the user to take an active role within the RLOs via activities and self-assessment aligned to these learning goals (Biggs & Tang 2003). RLOs can be adapted to specific patient groups, constitute only a small amount of learning time, and can be replayed until the knowledge or skill has been mastered. Furthermore, RLOs have been shown to improve motivation and compliance of treatments in clinical groups (Murray et al. 2001). Video materials for hearing aid users have been developed previously to supplement standard hearing aid care (e.g., Kramer et al. 2005). However, the development of materials using the underpinning theoretical principles of the RLO concepts as described above, with the inherent range of multimedia that were developed by using an iterative participatory approach, have not been used with hearing aid users. As hearing aid follow-up appointments are not routinely offered in the UK, all the important information needs to be given at the time of the hearing aid fitting. As such, RLOs offer advantages in that they can provide essential and supplementary information, be used at a time that suits the patient, used as many times as the user wants and needs, and can be delivered remotely in the home environment.

Internet delivery of educational programs, along with other teleaudiology applications, has the added potential of being accessible to many people, including hard-to-reach populations, provided they have access to the internet (Swanepoel & Hall 2010). While there is an increasing use of internet-based solutions to deliver educational resources, one current logistical problem in the internet delivery of patient education and support to first-time hearing aid users is that internet use is relatively low in this age group. We reported on PC and internet use in a random sample of 1298 UK 50- to 74-year-olds (Henshaw et al. 2012). In the typical first-time hearing aid user age group (70 to 74 years), PC and internet use was significantly lower (36.4 and 17.5%), than the youngest age group (50 to 54 years) where use was 84.6 and 65.5%. Thus, to maximize accessibility of educational programs in the first-time hearing aid user population, it is important to consider a range of delivery methods, such as DVD for TV and PC, and internet.

We aimed to develop and evaluate a series of RLOs for first-time hearing aid users, based on guidance on the development, evaluation, and implementation of complex interventions provided by the Medical Research Council (2008). This guidance suggests that interventions should be developed systematically and includes four key stages. *Development* of an intervention should be based on an identified evidence base and an underlying theory. *Feasibility* of the intervention informs recruitment and retention of participants, delivery, accessibility and take-up of the intervention, in addition to acceptability and adherence of participants with the intervention. The *evaluation* phase assesses the effectiveness of the intervention, ideally by an RCT to prevent selection bias, where selection of appropriate outcome measures is crucial. The final stage, *implementation*, ensures that the intervention translates into clinical practice and assessment of long-term outcomes, to show whether the intervention can generalize to wide-scale implementation and effectiveness.

The specific aims of this research were to:

1. develop a series of short interactive RLOs to cover a broad range of auditory rehabilitation content, both practical and psychosocial, which could be accessible to hearing aid users and their family and friends;
2. establish accessibility, take-up, acceptability, and adherence of the RLOs;
3. assess the benefits and cost-effectiveness of the RLOs in first-time hearing aid users.

## METHODS

This study is reported in accordance with the CONSORT statement (Schulz et al. 2010) that offers guidance for the transparent and unbiased reporting of RCTs.

### Participants

Adult first-time hearing aid users were recruited via the Nottingham Audiology Service at the Nottingham University Hospitals NHS Trust who had been referred for hearing assessment by their family doctor. Of the 553 patients who attended the assessment appointment and had not previously worn hearing aids, 203 (36.7%) were willing to take part and met the inclusion criteria. These were (1) adults ages ≥18 years, (2) first-time hearing aid users, and (3) spoken English as first language or good understanding of English. Exclusion criteria were those who were unable to (1) access DVD, PC or internet, or (2) complete questionnaires due to age-related problems.

### Study Design and Procedure

The design was a prospective, registered clinical RCT with two arms. The intervention group (RLO+, n = 103) received the RLOs plus standard clinical service including hearing aid(s) and counseling, and the waitlist control group (RLO−, n = 100) received standard clinical service only. Patients who met the eligibility criteria at the hearing assessment appointment were asked by the clinical audiologist if they were interested in participating in the study. Details of the study, including randomization and offer of the RLOs to the control group postevaluation, were explained both verbally and in the participant information sheet. Informed, written consent was obtained by the audiologist at the hearing aid fitting appointment approximately 4 weeks later to meet the ethical requirement of at least a 24-hr consideration period. Eight experienced clinical audiologists received training in the consent procedure, as well as how to use and demonstrate the RLOs as part of the study protocol by MF and MB.

Postconsent, participants were allocated to one of two groups. Allocation was based on a computer-generated pseudo-random code using random arrangement of blocks of randomly varying size, created by the Nottingham Clinical Trials Unit to provide a robust randomization method. Allocation to groups was in the ratio of 1:1, stratified by age (<70 years, ≥70 years), and made by the recruiting clinical audiologist who accessed a remote-controlled randomization system managed by the Nottingham Clinical Trials Unit. Allocations were revealed to the research team on completion of the study.

Those who were allocated to the RLO+ group chose their preferred RLO delivery method from one of four options: (1) interactive DVD for television, (2) interactive DVD for PC, (3) interactive RLOs via the internet, and (4) autoplay DVD for television with no interactivity. The DVD was given at the hearing aid fitting appointment, and access to the internet was given within 1 to 2 days of receiving the hearing aid. Participants were requested to watch all the RLOs, preferably no more than two per day to minimize information overload.

Participants attended an evaluation session approximately 5 to 8 weeks postfitting (M = 6.8 weeks, SD = 1.2, range 4 to 12, 91% seen between 5 and 8 weeks). The research audiologists (n = 2) were blinded as to whether the participants had received the RLOs or not. Before attending the evaluation session, the participants were requested not to reveal their group until the end of that session, to minimize researcher bias in outcome measurement. On completion of the evaluation session, the RLO− group was offered the RLOs although there was no further evaluation. The primary outcome measure was hearing aid use measured by the Glasgow Hearing Aid Benefit Profile (GHABP, Gatehouse 1999). On the basis of an improvement of 12.5% use, equivalent to a half-category increase on the response scale of the GHABP, and based on 80% power and a two-sided type I error rate of 5%, 85 patients were required for each group. To allow for an estimated 15% dropout rate, a total recruitment of 200 patients was planned.

The study was approved by the Nottingham Research Ethics Committee and Nottingham University Hospitals NHS Trust Research and Development department. Participants were paid a nominal inconvenience fee and travel expenses to attend the evaluation session.

### Intervention

#### RLOs

RLOs were developed using a participatory, community of practice approach, and were based on pedagogical design principles (Biggs & Tang 2003; Koper 2003; Windle et al. 2010) described in more detail elsewhere (Ferguson 2014; Ferguson et al. 2015). In brief, a Delphi review of 33 hearing healthcare professionals identified by consensus the key informational elements, including topic areas, for inclusion in the RLOs (Ferguson et al. 2012). These then informed two workshops of 35 hearing aid users (compliant and noncompliant) and one workshop of 11 audiologists, where RLO content was developed based on their personal experience and insights. The RLOs were designed to be delivered at the lowest technological fidelity (i.e., DVD) to maximise accessibility, as previous research had shown that PC and internet use in the typical first-time hearing aid user age group was relatively low (Henshaw et al. 2012). The DVD could also be used with television and PC, and RLOs were also available via the internet through the Nottingham Hearing Biomedical Research Unit internet portal.

There were seven RLOs (getting to know your hearing aids; how to insert hearing aids; what to expect when wearing hearing aids; adapting to wearing hearing aids; communication tactics; using the phone and other devices; hearing aid care and troubleshooting), with duration between 4 and 11 min (M = 7.89 min, SD = 2.5). There was also a short introduction (2.8 min), and altogether the total duration of the RLOs was 58.7 min. Sample RLO clips can be found in the videos in Supplemental Digital Content 1 (http://links.lww.com/EANDH/A236). The RLOs were based on pedagogical principles, introducing specific learning outcomes at the outset, reinforcement of good behaviors, and explaining the consequences of poor behaviors, with an interactive multiple-choice quiz at the end of each RLO so the user could see what they had learned. The RLOs included video clips, illustrations, animations, photos, sounds and testimonials, and all were subtitled. The importance of psychosocial aspects of hearing loss was evident from the Delphi review and workshops. Therefore, aspects such as emotions, confidence, and involvement of friends and family members were included in the Expectation, Adaptation and Communication RLOs.

The interactive nature of the RLOs included (1) choice of ear mold (custom or open, as some of the RLOs were ear mold specific, e.g., how to insert hearing aids); (2) choice of RLOs from a user-friendly interface; (3) the option to rewind, fast-forward, and pause; and (4) the interaction with a two- or three-question multiple-choice quiz, based on a three-option, three-alternate forced-choice paradigm, after each RLO via the TV remote control handset or PC mouse. After selecting the answer, the participant was shown whether this was correct or not, along with some additional advice, before moving onto the next question. Participants were able to replay the quiz as many times as they wished. An autoplay version was developed for those unable to use a TV remote control handset. Using a platform for DVD delivery meant that the participants’ interactivity with RLOs was limited compared with that for RLOs developed specifically for internet delivery. However, given the low PC and internet use in the typical first-time hearing aid user group, we decided the compromise between lower interactivity and higher accessibility for typical first-time hearing aid users was acceptable.

### Hearing Aids

Hearing aids (Oticon Zest, Phonak Nathos) were fitted, programmed using the NAL-NL1 algorithm and verified by real-ear measurement in accordance with local protocols and national guidelines (British Society of Audiology 2008). Hearing aids were fitted with either custom ear molds or open-fit slim tubes and the volume control (VC) was routinely deactivated.

### Outcome Measures

#### Audiological Measures

Pure-tone air conduction thresholds were measured at octave frequencies (0.25 to 8 kHz) for each ear, and bone-conduction thresholds as required (0.5 to 4 kHz), following the procedure recommended by the British Society of Audiology (2011), using a Siemens Unity PC audiometer (Crawley, West Sussex, UK) and B71 audioear (New Eagle, PA).

#### Self-Report Questionnaires

All questionnaires were completed by interview at the evaluation session unless specified otherwise.

The *GHABP* (Gatehouse 1999) assesses hearing disability (or activity limitations) and handicap (or participation restrictions; part 1), and hearing aid use, benefit, residual disability and satisfaction (part 2). Each domain is measured on a five-point scale, and the mean score across the four predefined situations was converted into a percentage. Hearing aid use was the primary outcome measure. Part 1 was completed before the hearing aid fitting by the clinical audiologist, and parts 1 and 2 were completed for the aided condition at the evaluation session.

The *Practical Hearing Aid Skills Test (PHAST*; Desjardins & Doherty 2009) tests eight hearing aid skills (insertion, removal, opening battery door, changing the battery, cleaning the aid, manipulating the VC, telephone use, and use of programs). The VC skill was not tested as VC was routinely deactivated. Each skill was scored on a five-point Likert scale (0 = cannot perform, 4 = excellent). Results are expressed as percentages.

The *Satisfaction with Amplification in Daily Life (SADL*; Cox & Alexander 1999) is a 15-item questionnaire, from which four composite scores are derived (positive effect, service and cost, negative features and personal image). Each item is scored using a seven-point Likert scale (A = not at all to G = tremendously). Question 7 was altered to say “Are you bothered by an inability to get enough loudness from your hearing aids?” Questions 14 and 15, which consider the cost and dependability of hearing aids, were excluded as all hearing aids were provided free of charge by the UK National Health Service.

The *IOI-HA* (Cox & Alexander 2002) is a seven-item questionnaire (use, benefit, residual activity limitation, satisfaction, residual participation restriction, importance to others, quality of life) and is scored on a five-point scale, where a high score indicates greater benefit.

The *HHIE* (Ventry & Weinstein 1982) is a 25-item questionnaire designed to assess the effects of hearing loss on the emotional (n = 13), social and situational adjustment (n = 12) of older people, scored using a three-point scale (4 = yes; 2 = sometimes; 0 = no). The questions were asked as though the participants were wearing their hearing aids.

The *Hearing Aid and Communication Knowledge* (*HACK*; Ferguson et al. 2015) is a 20-item open-ended questionnaire that measures free recall of knowledge relevant to practical (n = 12) and psychosocial (n = 8) issues on hearing aids and communication. For each item, there are two or three predefined answers, and one point was given for each response that met the predefined answers. For example, the question “How frequently, and when, does the tubing need to be replaced in the ear mold?” has the following predefined answers: (1) every 4 to 6 months, and (2) when the tubing becomes worn or damaged (e.g., yellow, hard, or split), with a maximum score of 2. The scores for the practical (max = 32) and psychosocial (max = 22) scales, and a combined total score are presented as percentage correct from the total number of possible correct answers.

The *Hospital Anxiety and Depression Scale* (*HADS*; Zigmond & Snaith 1983) is a 14-item scale designed to assess psychosocial well-being, specifically levels of anxiety (n = 7) and depression (n = 7). Each item is scored from four possible responses (0 to 3), where a high score indicates greater anxiety and depression. The HADs was completed postfitting at home and at the evaluation session.

The *Short Form Patient Activation Measure* (*PAM*; Hibbard et al. 2005) is a 13-item measure that assesses patient knowledge, skill, and confidence for self-management of their health. Each item is scored on a four-point ordinal scale (0 = disagree strongly to 3 = agree strongly). The PAM was completed postfitting at home and at the evaluation session.

The *EQ-5D* (Euroqual 1990) is a standardized measure of health status that provides a generic quality of life measure used in the clinical and economic evaluation of health care. There are five dimensions: mobility, self-care, usual activities, pain/discomfort, and anxiety/depression, rated as one of three levels (no, some, or extreme problems). In addition, there is a visual analogue scale that records self-rated health on a 0 to 100 scale (0 = worst imaginable health state; 100 = best imaginable health state). The EQ-5D was completed at the fitting (baseline) and evaluation (follow-up) appointments.

*IT literacy* was based on a computer skill scale (never used a computer, beginner, or competent) that has been previously validated (Henshaw et al. 2012).

*Hearing aid use* (average hours/day) using data logging information integral to the hearing aid was obtained for each participant for the period between the fitting and evaluation appointments.

#### Participant Feedback

The *Video Diary* recorded how often and when (i.e., dates) a participant viewed each RLO, and how useful each RLO was (0 = no use at all, 10 = extremely useful). Participants were instructed to watch all the RLOs, preferably across several days, and to concurrently note their comments.

The *RLO Feedback Questionnaire* was adapted from a questionnaire from the RLO Center for Excellence in Teaching and Learning toolkit (CETL 2009), which assessed participant feedback on the RLOs using quantitative and qualitative measures. These included 17 statements for which participants were asked to rate their agreement on a five-point Likert scale (1 = strongly disagree to 5 = strongly agree). Open-ended questions asked about the best and worst aspects of the RLOs. Finally, descriptor words that described the usability and desirability of the RLOs were selected from 60 options (e.g., rewarding, motivating, stressful; Benedek & Milner 2002). Participants were asked to choose all the words that were relevant to their experience, and then to identify the top five words that best described their experience.

*Focus Groups* (n = 3) were led by two authors (PL and MF), and included participants from the RCT (n = 20) and their communication partners (n = 5). The aims were to obtain feedback on the RLOs in terms of content, how they were used and how they affected participants’ hearing and communication. In addition, topic ideas and feedback for future RLO development was sought. The focus groups were recorded, transcribed, and analyzed using thematic analysis according to Braun and Clarke (2006).

### Analysis of Outcome Measures

Planned analysis initially examined the difference between groups for the primary outcome measure (GHABP hearing aid use) and the secondary outcome measures obtained at evaluation, using either an independent *t* test or Mann–Whitney test. Group means (or median) and the 95% confidence intervals were presented as planned. For significant results, an adjusted analysis using multiple linear regression, adjusting for age, gender, and hearing loss (better-ear average [BEA] for 0.25 to 4 kHz) was performed. Bonferroni correction to account for multiple comparisons was applied where necessary. For outcome measures obtained at both fitting and evaluation (HADS and PAM), an analysis of variance was performed with “group” and “visit” as factors. Mode of RLO delivery was not a covariate. Effect size (Cohen’s *d*) was categorized as small (0.2), moderate (0.5), and large for (0.8). Significance was set to *p ≤* 0.05.

For the cost-effectiveness analysis, responses from the EQ-5D were transformed into a quality-adjusted life year (QALY) score using the scoring metric as described by Dolan (1997). Incremental QALYs were calculated as the difference between the baseline and follow-up QALYs for the two groups. Total cost per participant was calculated as the average cost of specific activities within the patient pathway, and the incremental cost was the cost difference between the two groups (i.e., the costs of the RLOs, which were costed at £2 per set). The incremental cost-effectiveness ratio (ICER) was the incremental QALY divided by incremental costs. In the UK, the willingness to pay per QALY is an arbitrary threshold value set by the National Institute for Health and Care Excellence, which is typically set at an ICER of £20,000 per QALY (NICE 2013).

## RESULTS

### Participants

Demographic and clinical characteristics of those who (1) were initially assessed, (2) were eligible and consented at the fitting appointment, and (3) participated in the evaluation session are shown in Table 1. There were no significance differences between the RLO+ and RLO− groups for age, gender, hearing (BEA across 0.25 to 4 kHz), IT literacy at the fitting or evaluation appointments. There was, however, a highly significant difference (*p* < 0.001) for age, gender, and hearing loss between those who participated in the RCT (P+) and those who had a hearing test but did not participate (P−). Age was lower in the P+ group (P+, M = 67.9 years, SD = 9.5, range = 42 to 95; P−, M = 73.9, SD = 11.6, range = 27 to 95), there were fewer females in the P+ group (P+, females = 41.4%: P−, females = 57.6%) and the BEA was better (P+, M = 32.8 dB HL, SD = 8.8; range = 6 to 59: P−, M = 39.5, SD = 11.1, range = 12 to 74) than in the P− group.

**TABLE 1.**
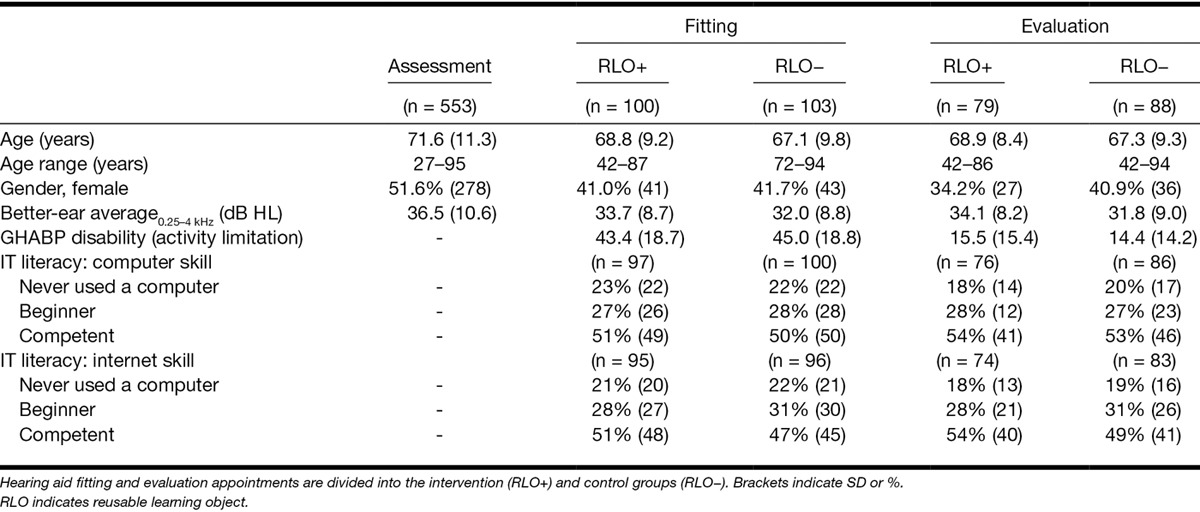
Mean demographic and clinical characteristics of those who attended the (1) hearing assessment appointment, (2) hearing aid fitting appointment, and (3) evaluation appointment

### Access, Take-Up, and Adherence

Of the 553 patients who attended the hearing assessment appointment who had never worn hearing aids, 370 (66.9%) met all the eligibility criteria, and of those 203 (54.9%) consented to participate in the study. The numbers recorded who did not meet the eligibility criteria were poor understanding of English, n = 32 (5.8%), unable to access a DVD, PC, or the internet, n = 116 (20.9%), unable to complete the questionnaires, n = 53 (9.8%), or fully understand the study requirements, n = 40 (7.2%; e.g., due to age-related cognitive decline or dementia). Of the four delivery formats used, DVD for TV was the most commonly used (50.6%), followed by internet delivery (32.9%), DVD for PC (15.2%), and DVD autoplay (1.3%).

Of those who were eligible to participate at the hearing assessment, 290 (78.4%) expressed an interest in participating in the study at the hearing assessment. At the fitting appointment, one person who was eligible declined to consent because they were not interested in watching the RLOs. Adherence in those who attended the evaluation session and watched the RLOs was very high (n = 77; 97.4%), with only two participants failing to watch any of the RLOs. Video diaries were completed by 71 (89.9%) participants, and of those, 67 (94.3%) participants watched all seven RLOs (67% of all those who received the RLOs). Of the four participants who did not watch them all, two participants watched five RLOs and two participants watched three RLOs, one of whom had difficulties accessing the RLOs online as he used the Linux operating system. The average number of views per participant was 13.0 (SD = 7.1, maximum = 36). On average, around half (49.2%) the participants watched the RLOs at least twice, and around a fifth (22.1%) watched the RLOs at least three times, with some participants watching the RLOs as many as seven times (Table 2). This reuse of RLOs suggests that the RLOs were being used by the participants to self-manage their hearing loss, hearing aids, and communication. Sixty-two (78.4%) participants said they would recommend the RLOs to other people.

**TABLE 2.**
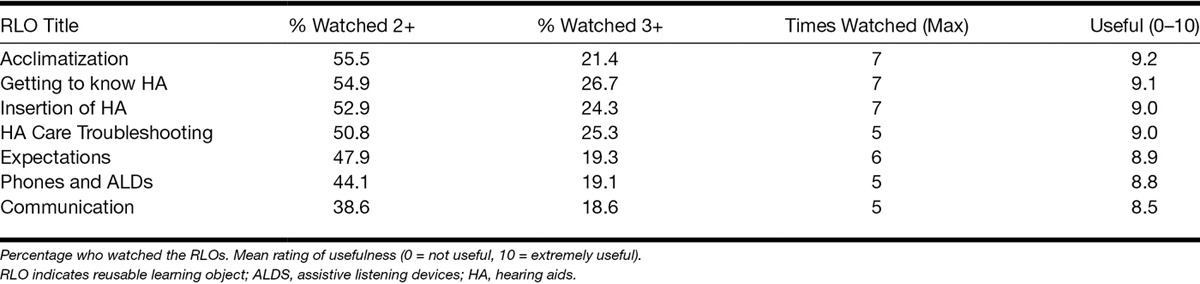
RLO reuse suggests self-management

Attrition between the fitting and evaluation sessions was slightly higher than anticipated at 17.8% (n = 39), and slightly more for the RLO+ group (20.3%) than the RLO− group (15.0%). There was no significant difference in age and hearing loss between those who attended and did not attend the evaluation session (age; attended, M = 68.1 years, SD = 8.9, nonattenders, M = 67.3 years, SD = 12.1: BEA; attended, M = 32.0 dB HL, SD = 8.7, nonattenders, M = 32.7 dB HL, SD = 9.2). There was, however, a significant difference in gender, with more women (28.4%) dropping out at evaluation session than men (12.6%; *p* = 0.02). Reasons given for nonattendance were health problems (n = 8), personal not study related (n = 5), poor mobility (n = 2), husband ill/died (n = 2), could not take time off work (n = 2), no longer wishes to take part (n = 2), hearing better, no longer wears aids (n = 1), no reason obtained (n = 17, 43%).

### Outcome Measures

Table 3 shows the outcome measures by group. There was no between-group difference in the primary outcome measure, overall GHABP hearing aid use (Mann–Whitney *Z* = 0.80, *p* = 0.48). Similarly, there was no group difference for hearing aid use measured by data logging [*t*(152) = 0.95; *p* = 0.34].There were significant differences between the two groups for overall practical hearing aid handling skills (PHAST; Mann–Whitney *Z* = −3.7; *p* < 0.001), and overall knowledge of hearing aids and communication issues [HACK, *t*(139) = 9.3; *p* < 0.001]. These remained significant after Bonferroni correction. None of the other outcome measures showed a significant between-group difference.

**TABLE 3.**
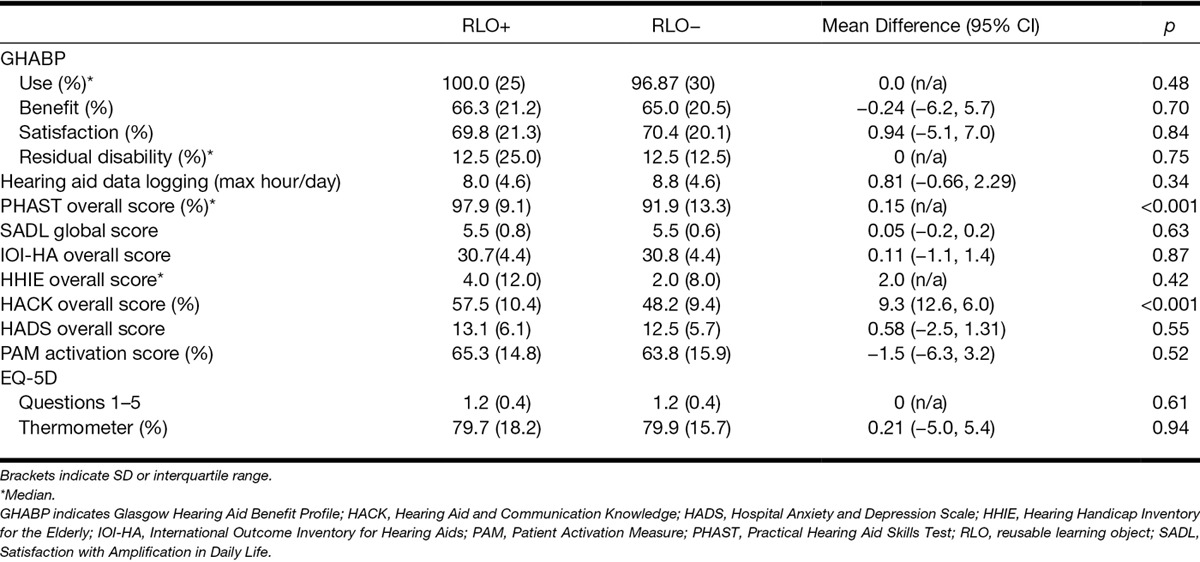
Mean outcome measures for the intervention (RLO+) group and the control (RLO−) groups at evaluation

**Fig. 1.**
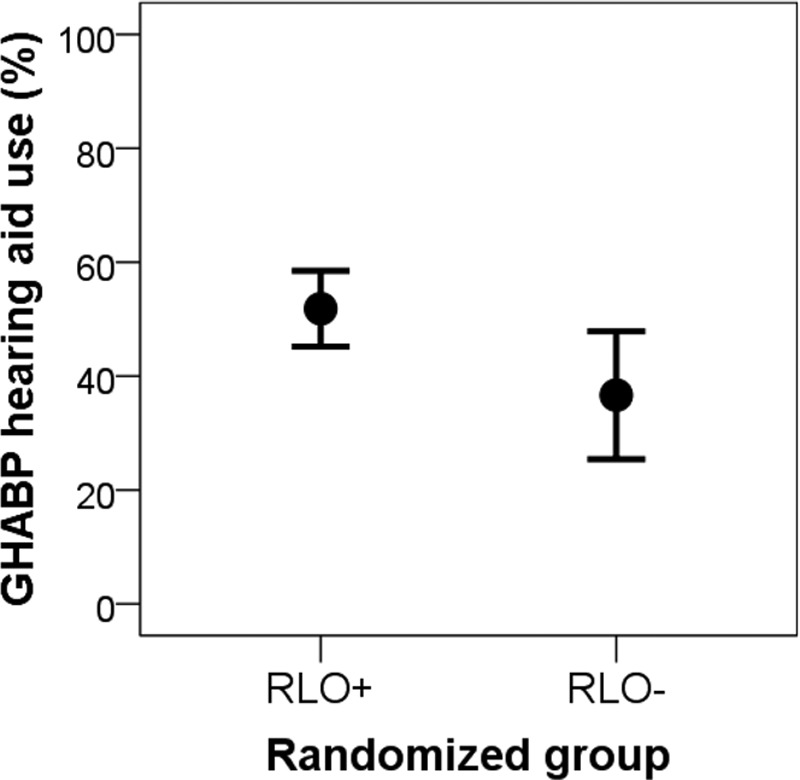
GHABP hearing aid use in the suboptimal users (GHABP use < 70%). Mean ± 95% confidence interval for the intervention (RLO+; n = 17) and control (RLO−; n = 22) groups. GHABP indicates Glasgow Hearing Aid Benefit Profile; RLO, reusable learning object.

Secondary analysis assessed for hearing aid use (GHABP) in suboptimal hearing aid users. There is no consensus in the literature on what constitutes suboptimal use, so we chose GHABP use <70%, which was approximately the mean use from a large sample of hearing aid users who had hearing loss in the better ear at 30 to 39 dB HL (Whitmer et al. 2014). There was a significant improvement in hearing aid use for suboptimal hearing aid users [*t*(37) = −2.3; *p* = 0.03], where the RLO+ group used their hearing aids on average 15.2% more than the RLO− group (RLO+; M = 51.8%, SD = 12.9; RLO−; M = 36.6%, SD = 22.3), with a large effect size (*d* = 0.83; Fig. 1). Similar significant results were seen for suboptimal use defined as between <75 and <50% in 5% steps. Although a significant increase in hearing aid use was shown for the predefined situations “having a conversation in a group” and “carrying on a conversation in a busy street or shop” for the RLO+ group compared with the RLO− group (Fishers test, *p* = 0.02 and *p* = 0.05, respectively), these results were no longer significant after applying Bonferroni corrections. There were 5 (5.6%) hearing aid nonusers in the RLO− group compared with none in the RLO+ group.

Table 4 shows the PHAST subscales in terms of the median, interquartile range, and the percentage of participants who achieved a 100% score for each group. The RLO+ group showed significantly better handling skills for the Telephone (Mann–Whitney *Z* = −3.4; *p* = 0.001) and Cleaning Ear mold domains (Mann–Whitney *Z* = −2.84, *p* = 0.005). These remained significant after Bonferroni correction. There was no significant difference between the groups for hearing aid insertion/removal (*Z* = −0.067, *p* = 0.95) and battery door/change (*Z* = −0.86; *p* = 0.39). A quantile regression analysis of PHAST overall score, accounting for age, gender, and BEA, showed there remained a significant effect of group (*p* = 0.002) and there was a significant effect of age (*p* = 0.04), where older people had poorer practical skills, but no effect of gender (*p* = 0.10) or BEA (*p* = 0.11).

**TABLE 4.**
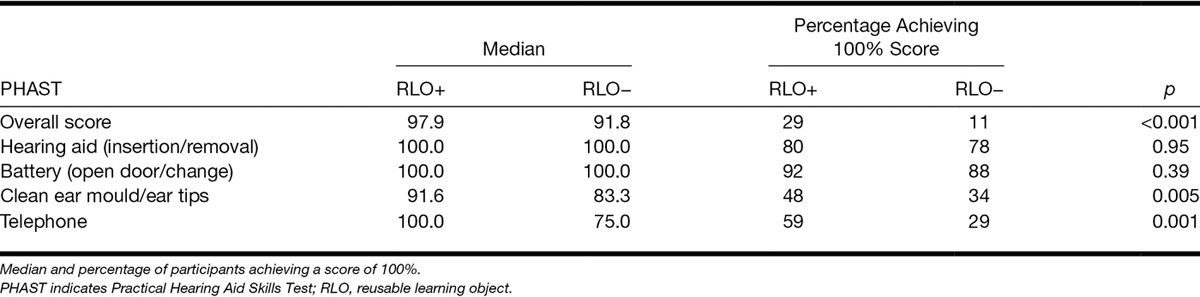
PHAST results for the overall and subscale scores

**Fig. 2.**
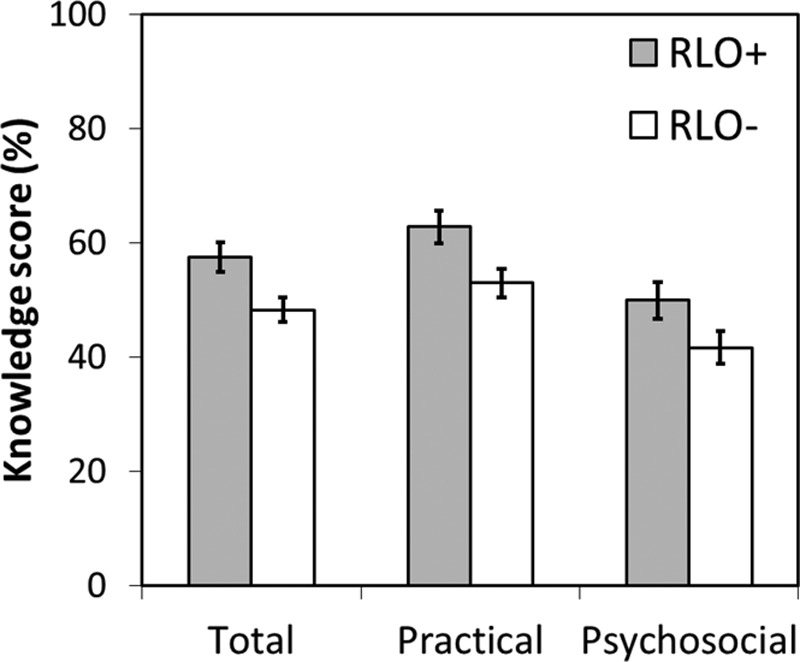
HACK score. Mean ± 95% confidence interval for the intervention (RLO+) and control (RLO−) groups. HACK indicates Hearing Aid and Communication Knowledge; RLO, reusable learning object.

For the HACK questionnaire, the RLO+ group had significantly better knowledge than the RLO− group at 6-weeks postfitting for the overall score [*t*(139) = 5.5, *p* < 0.001, *d* = 0.94], and both the practical [*t*(139) = 5.14, *p* < 0.001, *d* = 0.88] and psychosocial questions [*t*(139) = 3.9, *p* < 0.001, *d* = 0.65; Fig. 2]. These remained significant after Bonferroni correction. Knowledge was better for practical issues than psychosocial issues in both groups, with large and moderate effect sizes, respectively. After accounting for age, gender, and BEA, there remained a highly significant effect of group (*p* < 0.001) for the overall score, with no effect of age, gender, and BEA. More detailed results can be found in Ferguson et al. (2015).

There was no significant effect of group for the remaining outcome measures obtained at the evaluation session (Table 3). The HADS and PAM were obtained at fitting and evaluation. For the HADS, there was no significant main effect of group [*F*(1, 316) = 0.031, *p* = 0.86] nor visit [*F*(1, 316) = 1.55, *p* = 0.21] nor any significant interaction. For the PAM, there was no significant main effect of group [*F*(1, 338) = 0.13; *p* = 0.72] nor visit [*F*(1, 338) = 0.31; *p* = 0.58] nor any significant interaction.

### Participant Feedback on RLOs

Table 5 shows number and percentage of RLO+ participants who either agreed, disagreed, or neither agreed or disagreed, with the 17 statements about the RLOs, ranked by positivity of the statements. Three-quarters or more were positive about 15 (88%) of the statements, suggesting there was favorable and positive feedback on the RLOs. For *content*, the vast majority (>92%) agreed the illustrations and videos aided their understanding and the RLOs held their interest. For *activity and engagement*, 91% agreed the interactive quiz gave a clear message, and 88% would refer back to RLOs if they had a problem. For *self-assessment*, there was high agreement (>88%) that the quiz was valuable and gave clear messages as to what was right and wrong.

**TABLE 5.**
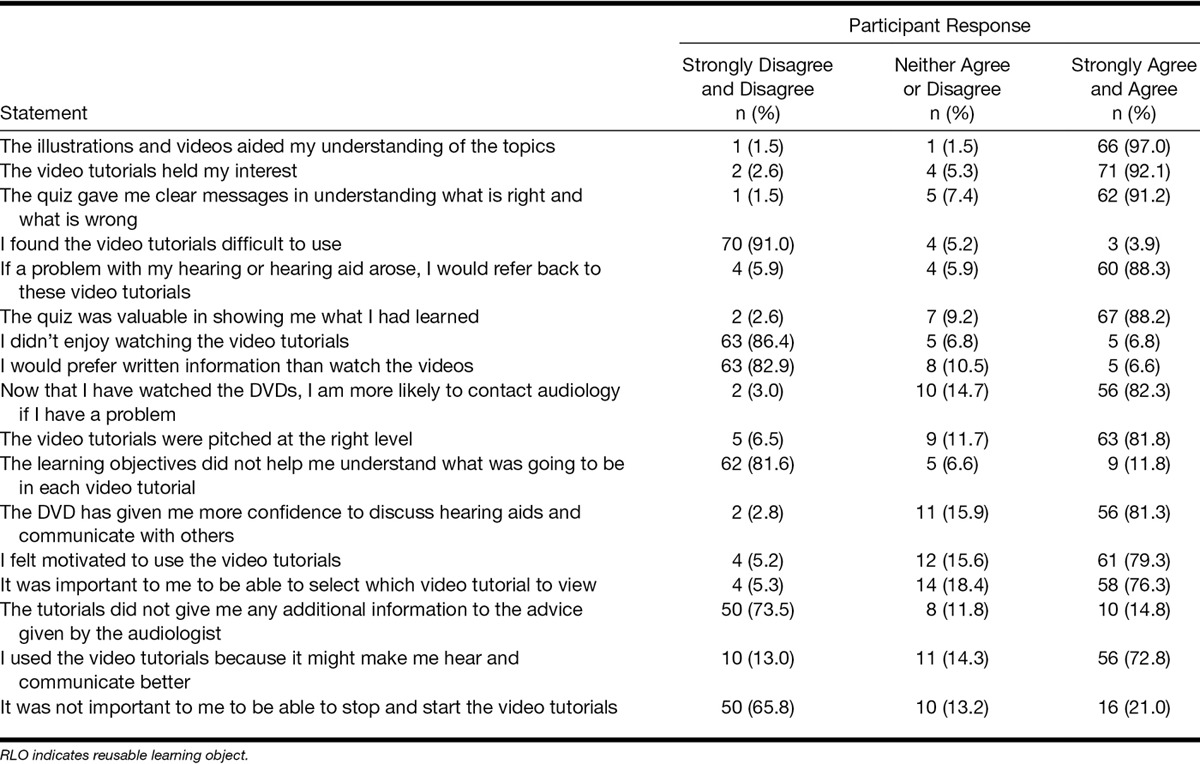
RLO feedback questionnaire statements ranked in order of positivity

**Fig. 3.**
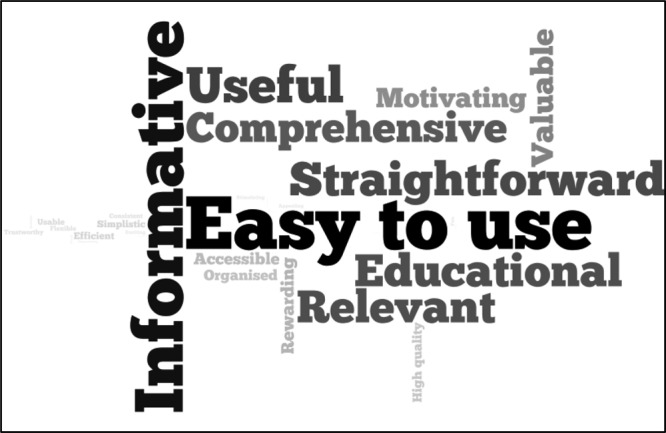
Wordcloud to show the top five words to describe the reusable learning objects. The larger the font size, the more frequently the word was selected.

In addition, the top five key words chosen by each person from a list of 60 words to describe the RLOs are shown in the word cloud in Figure 3, where the larger the word the greater the number of participants chose that word. The top five chosen words were “easy to use” (58.2%), “informative” (53.2%), “useful” (39.2%), “straightforward” (36.7%), and “educational” (35.4%). The RLOs were rated as highly useful (M = 8.9, SD = 0.22; Table 2).

Three focus groups, with between 7 and 10 participants, were held comprising (1) 20 people who had participated in the RCT, and were representative of the RCT sample (age: M = 68.8 years, SD = 5.7, range = 42 to 94: BEA: M = 30.7 dB HL, SD = 7.1, range = 6 to 53; gender: 8 females, 40%), and (2) five communication partners (spouses, n = 3; daughters, n = 2). The key themes from the thematic analysis of the postevaluation focus groups were that the RLOs were aligned to people’s experiences, and the content was supported by the vast majority.

I did find it very helpful especially in areas when they talked about sounds and how loud things were because that quite shocked me at first, but then watching the video I found that was a normal reaction…

*I think the information they provided was fantastic. I got everything I wanted*.

*…I have watched it and I found it very useful. The first time was little tips on how to put hearing aids in, that was good. One interesting thing was how your brain learns what is loud and what isn’t loud and adjusts accordingly*.

Although not everyone was positive about the RLOs.

*I don’t think there was so much need to prolong everything so much and to talk as though we still couldn’t hear*.

*My daughter said it was very long…you could have put what they said in three minutes, not six minutes*.

Other themes included:

1. repeated watching of RLOs

… I found that I had missed something. So I went back and looked at it again,

2. sharing of videos with others, such as family, friends, and neighbors

I have passed my DVD on to an old couple who both have hearing aids … I kept telling her, Play that DVD and you will know why, because you have got to get used to it, haven’t you?

3. the RLOs provided reassurance, helped people to remember things and improved awareness and confidence

It [the DVD] explained how we have to learn to rehear things. That is not an aspect that I [was aware of], to re-educate the brain to interpret what you hear.

4. involvement of family members and friends

Well, I went through them, right the way through. I begged my wife to watch them as well which I thought was important.

I sat my husband down. I said, I would like you to watch this. Well he said, What do I need to watch this for? I am not deaf, but I said, It will help you to understand me. So he did…. yes, I did find it very, very useful for that.

### Health Economic Analysis

Table 6 reports the mean total unadjusted QALYs at baseline and 6-week follow-up, and incremental QALYs. There was no significant difference between control and intervention groups for both baseline (*p* = 0.91) and 6-week follow-up QALYs (*p* = 0.25) nor for incremental QALYs (*p* = 0.68). Using costs of the service from the healthcare purchaser’s perspective, there was a significant difference in total costs between both the control and intervention groups of −£0.90 (*p* < 0.0001). The mean ICER was −£479.41 (95% CI = −£20784 to £16155), indicating the DVD intervention was dominant over usual care (as it was more effective and cheaper). At a willingness to pay of £20,000 per QALY (NICE 2013), 70% of cases were cost-effective; however, the 95% confidence intervals suggest that there is some uncertainty in the results.

**TABLE 6.**
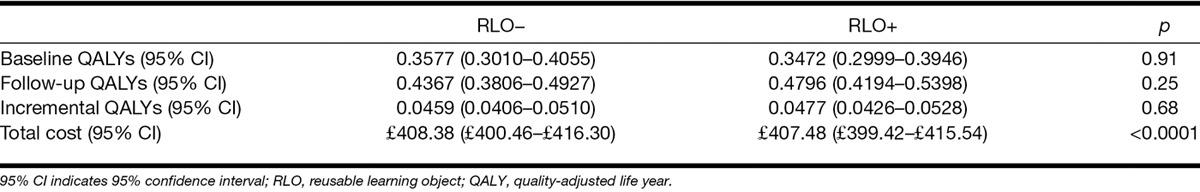
Results of the cost-effectiveness base case analysis

## DISCUSSION

The main aims of this research were to develop a series of interactive educational resources (RLOs) for first-time hearing aid users, assess the feasibility of delivering the RLOs to those attending hearing aid fitting appointments in an audiology clinic, and then evaluate the effectiveness of the home-delivered RLOs using an RCT, based on a framework for developing and evaluating complex interventions (Medical Research Council 2008).

### Development of RLOs

RLO content was developed using pedagogical principles from learning theory by taking a participatory approach that involved both hearing aid users and audiologists using a Delphi review, focus groups and workshops and iterative feedback during development (Ferguson et al. 2012; Brandreth et al. 2013). The participatory approach based on a validated methodology used in education enabled user perspectives and experiences to be incorporated into the RLOs (O’Keefe et al. 2008). Although this approach is labor intensive, it has been recognized that rich multimedia developed with high productions values has enormous power to engage learners and aid understanding (Edelson & Pittman 2001). The end product was seven high-quality production RLOs that included a range of multimedia to demonstrate concepts, meet specific learning objectives, and self-assessment of learning with an interactive quiz.

### Feasibility of Using RLOs in an Audiology Clinic Sample

To assess the feasibility of the RLOs as an intervention for first time hearing aid users, we measured participant recruitment and retention, delivery and accessibility, take-up, acceptability, and adherence of the intervention. *Recruitment* of the participants in the present study differed from other studies that have evaluated remotely delivered educational programs. These generally recruited existing users by advertising through the media thereby recruiting later in the patient journey as well as running the risk of sampling biases, such as self-selection and being better educated (Kramer et al. 2005; Lundberg et al. 2011; Thorén et al. 2011, 2014). It has been suggested that the timing of the delivery of educational support is most beneficial early on in the hearing aid user’s journey (Kramer et al. 2005).

By recruiting participants prospectively, we aimed to recruit a representative sample of the typical hearing aid clinic population. However, compared with those who presented for hearing assessment, our recruited sample were younger, had better hearing, and there were fewer women. It is likely that these significant differences resulted from the study eligibility criteria. For example, despite our best efforts to maximise accessibility to the RLOs, the main reason for nonparticipation was because a large number of patients, around one-fifth, could not access the RLOs as they did not have access to a DVD player, PC, or internet. Although we expected PC and internet use to be low (Henshaw et al. 2012), lack of access to DVD was poorer than expected given the percentage of households in the UK with DVDs in 2012 was 87% (Statista 2015).

*Delivery* and *accessibility* of RLOs using a solely online route was recognized at the outset as a barrier for many first-time hearing aid users, as we had shown previously that internet use in a large random sample (n = 473) of 65- to 74-year-olds was 26.2% (Henshaw et al. 2012). This was much lower than a Swedish study of 41 volunteers ages 65 to 74 years that showed 78% used the internet (Thorén et al. 2013). It is likely that these differences were due to the different volunteer recruitment criteria and methods. In the present study, internet use was higher than expected at 32.9%, similar to that of the 65- to 69-year age group (34%) reported by Henshaw et al., which reflected the average age of those who participated in the present study (M = 67.9 years).

Use of IT and smartphones is increasing in older adults (Deloitte 2014), with epidemiological data on internet use showing a year-on-year increase in 55- to 74-year-olds (2010 = 61%, 2012 = 70%, 2014 = 78%), suggesting that teleaudiology has the potential to become more prevalent in this age group (UNECE 2015). However, for the short term at least, there remains a digital divide due to age (Friemel 2014). To maximize accessibility and reduce barriers to educational resources for first-time hearing aid users, we propose that educational materials are developed across a range of flexible learning resources to future-proof the RLO concept. For those who have poor IT literacy, one option is to adapt the educational materials for a technology-free interactive booklet, although it should be noted in our present study that the majority (82%) preferred the RLOs to written information. Another option would be to take advantage of rapid technological developments that do not require IT skills but still allow interaction with RLOs, such as video e-cards. These are cardboard cards that can display graphics and sound via a small LCD (liquid–crystal display) screen. For those with good IT literacy skills, the RLOs could be adapted from the current “one-size-fits-all” approach, into shorter chunks or “bytes” of information to enable individualized tailoring to maximise relevance to individuals, with delivery capability increased to include mobile technologies (e.g., smartphones, tablets).

*Take-up*, estimated at 78%, was not measured directly but was assessed from those who were eligible and agreed to participate in the study. It is possible that without the additional research component the RLO take-up could have been even higher. In a service evaluation of the Nottingham Audiology Service where the RLOs in DVD format were paid for by the local NHS healthcare commissioning group and offered as part of standard care, the real-world take-up was indeed higher, at 90% (99/110). Seven people did not have the means to play the DVD, three did not want the DVD, and one could not understand English.

*Acceptability* of the RLOs was high, as demonstrated by take-up, ratings of usefulness, usability and desirability, the feedback questionnaire and postevaluation focus groups. The highly useful RLO rating (8.9/10) was consistent with the usability descriptors where “useful” was listed in the top three choices. There was high agreement on the feedback questionnaire for all three RLO components (content, activity and engagement, self-assessment). Practically, the RLOs were not difficult to use and were pitched at the right level. In terms of impact on their everyday lives, participants agreed they would be more likely to contact Audiology, and the RLOs gave them more confidence to discuss hearing aids and communicate with others. This suggests the RLOs were more than simply a tool to enhance information and knowledge in that they also had a positive impact on other activities and actions in their lives. Improved confidence has been suggested to be a critical element in the success of an intervention (Sweetow & Sabes 2010), and Kramer et al. (2005) reported their home-education program increased confidence in dealing with hearing loss. Similar responses were reported during the focus groups with themes of reassurance and improved confidence, as well as sharing the RLOs with others. Although the open-ended feedback questionnaire and focus groups showed favorable results, we do recognize that there is a potential for response bias as participants had invested time in the research (Kramer et al. 2005).

*Adherence* in the RLO+ group was extremely high, with only two participants reporting a failure to watch the RLOs. However, it remains a possibility that some nonattenders did not attend because they had not watched the RLOs. If we make this assumption (although many reasons for nonattendance were unrelated to the study, e.g., health reasons), only 67% watched all the RLOs. One of the interesting findings was that RLO reuse was considerable with at least half watching the RLOs two or more times, and some using them up to six to seven times. This reusability, which was also evident in the postevaluation focus groups, suggests that the participants were using the RLOs as a means to self-manage their hearing loss, hearing aids, and communication. This is an important finding as it is recognized in other health domains that patients who are motivated and actively participate in their care are more likely to adopt health behaviors that then lead to better patient outcomes (Mosen et al. 2007). This is particularly the case in patients with chronic conditions who are required to play a role in their day-to-day management, such as seen in those who have hearing difficulties.

It was notable that 78% said they would recommend the RLOs to others, and that sharing the RLOs with others and involving communication partners was a main theme from the focus groups. Most spouses report some degree of third-party disability (Scarinci et al. 2012), and the role of communication partners in the rehabilitation of people with hearing loss has been shown to be highly beneficial (Armero 2001; Stark & Hickson 2004; Scarinci et al. 2008). Therefore, the RLOs may act as a facilitator for encouraging discussion and a shared understanding of hearing and communication with others. Many who watched the RLOs felt more confident to discuss hearing-related issues, and there was a positive appetite in the focus groups for RLOs specifically for communication partners.

### Evaluation of RLOs

Evaluation of effectiveness of the RLOs using the RCT showed no significant group differences for the primary outcome measure (GHABP hearing aid use), although there were no nonhearing aid users in the RLO+ compared with the RLO− group (n = 5). However, for suboptimal users (GHABP hearing aid use <70%), there was significantly greater use in the intervention (RLO+) group compared with the control (RLO−) group. It could be argued that improvements in hearing aid use were shown where it mattered, which is when hearing aid use is lower than it could be. This suggests that if clinical resources are limited, RLOs are best targeted at those who are less likely to use their hearing aids optimally. Difficulties in noisy situations and in background noise are a major reason for nonuse of hearing aids (Kochkin 2000; Vuorialho et al. 2006; Bertoli et al. 2009; Linssen et al. 2013) and although there was greater use in the most complex listening situations (conversation in a group and busy street or shop), this was not significant after applying Bonferroni corrections.

There were benefits for the RLO+ compared with the RLO− group in terms of significantly better practical hearing aid skills and better knowledge on hearing aids and communication. The effect sizes were generally large, which is important when considering implementing an intervention into clinical practice as the clinical significance of the intervention is as important, if not moreso, than the statistical significance (Jacobson et al. 1984; Friedman et al. 2010; Sedgwick 2014).

For practical hearing aid skills, there was no group difference for hearing aid insertion/removal and battery functions, where both groups performed at ceiling. This probably reflects the importance audiologists place on ensuring that new users can manage the basics of hearing aid and battery insertion before leaving the clinic as an inability to do either renders the hearing aid virtually useless. Despite this, other studies have shown that difficulties with ear mold insertion and batteries are problematic in between 9 and 17% (Vuorialho et al. 2006; Bertoli et al. 2009; Desjardins & Doherty 2009; Goggins & Day 2009). Interestingly, the RLOs watched the most at 3+ times were Insertion and Getting to Know Your Hearing Aids, along with Hearing Aid Care and Troubleshooting. There were significant group differences shown in using the telephone and cleaning the ear mold and it is likely that these receive less attention from audiologists. Yet, the consequences of these are not trivial. Not being able to use the telephone with hearing aids is a major reason for hearing aid nonuse (Kochkin 2000) and the most common reason for repairs appointments is poorly maintained hearing aids (Block 2001), which also leads to hearing aid nonuse (McCormack & Fortnum 2013). In a 3-year follow-up of hearing aid users, 85% needed further explanation on how to use phones and the need for regular retubing (Goggins & Day 2009). Of note, is that although the participants in the present study only recently acquired their hearing aids, the practical hearing aid skills in the control group were on average 10% higher than those reported in experienced users (78.6%; Desjardins & Doherty 2009). This may be because age was a significant factor in both studies, with our participants having a lower mean age 67.9 compared with 75.3 years in the Desjardins and Doherty study. This highlights the importance of early fitting of hearing aids early to minimize the effect of poor handling skills (Davis et al. 2007).

Knowledge of psychosocial issues (e.g., how to improve communication) was significantly poorer than practical issues (e.g., frequency of tube replacement) in both groups. This suggests that either psychosocial issues receive less attention in hearing aid appointments or retention of knowledge of the more complex psychosocial issues is poorer. Either way those who received the RLOs had significantly better knowledge of hearing aids and communication 6-weeks postfitting, with large effect sizes, indicating that the RLOs facilitated improved knowledge and awareness of hearing-related issues. Furthermore, Lundberg et al. (2011) suggest that knowledge arising from educational guidance can increase confidence and set realistic expectations. It should be noted, however, that the mean total knowledge score in the RLO+ group of 58% and maximum score of 74% suggests there are still gaps in knowledge for most users. More research is needed to identify the impact of hearing-related knowledge on everyday life for new hearing aid users.

There were no between-group differences in the remaining hearing aid outcome measures (IOI-HA, HHIE, SADL). The results of the IOI-HA were similar to those reported in other studies of educational programs (Kramer et al. 2005; Lundberg et al. 2011; Thorén et al. 2011). However, Thorén et al. (2014) reported some improvement on two of the items (residual participation restriction and impact on others) in those who received the improved online intervention. It may be that the IOI-HA is not sufficiently sensitive to detect incremental benefits over and above that of the hearing aid. Similarly, there was no group difference on satisfaction (SADL) or psychosocial wellbeing (HADS), consistent with the other studies (Thorén et al. 2011, 2014; Lundberg et al. 2011). Some studies suggest longer term improvement in wellbeing becomes enhanced across time (Kramer et al. 2005; Thorén et al. 2014), whereas other benefits shown here, such as practical handling skills and knowledge, were revealed early on. It is notable that improvements in participation restrictions measured by the HHIE have been shown previously (Lundberg et al. 2011; Thorén et al. 2011, 2014), yet we showed no group differences. It may be that the RLOs do not reduce participation restrictions per se, or else the participants in the present study had fewer participation restrictions at the outset. Thorén et al. specifically targeted those with greater difficulties and this may have accounted for some differences.

There were no between-group differences in the remaining hearing aid outcome measures (IOI-HA, HHIE, SADL). The results of the IOI-HA were similar to those reported in other studies of educational programs (Kramer et al. 2005; Lundberg et al. 2011; Thorén et al. 2011). However, Thorén et al. (2014) reported some improvement on two of the items (residual participation restriction and impact on others) in those who received the improved online intervention. It may be that the IOI-HA is not sufficiently sensitive to detect incremental benefits over and above that of the hearing aid. Similarly, there was no group difference on satisfaction (SADL) or psychosocial wellbeing (HADS), consistent with the other studies (Thorén et al. 2011, 2014; Lundberg et al. 2011). Some studies suggest longer term improvement in wellbeing becomes enhanced across time (Kramer et al. 2005; Thorén et al. 2014), whereas other benefits shown here, such as practical handling skills and knowledge, were revealed early on. It is notable that improvements in participation restrictions measured by the HHIE have been shown previously (Lundberg et al. 2011; Thorén et al. 2011, 2014), yet we showed no group differences. It may be that the RLOs do not reduce participation restrictions per se, or else the participants in the present study had fewer participation restrictions at the outset. Thorén et al. specifically targeted those with greater difficulties and this may have accounted for some differences.

The RLOs were shown to be cost-effective in just over two-thirds of users. Such a finding with the EQ-5D is not common in hearing research as the EQ-5D is usually insensitive to hearing-related interventions (Chisolm et al. 2007). The relative gains in the incremental QALYs for the RLO+ compared with the RLO− group were very small, but because the cost of the DVD is very low (set at £2/DVD for the analysis presented), the RLO intervention was dominant over the standard care because it provided a more effective and cheaper pathway. It may well be that a health-related quality of life measure that is more sensitive to hearing interventions, such as the Health Utilities Index (HUI3; Davis et al. 2007), would provide more robust cost-effectiveness results. However, from a health commissioner’s perspective, the low cost of this intervention is unlikely to be a barrier to implement these RLOs into clinical practice.

The choice of outcome measures to assess the benefits of interventions is a major issue in adult rehabilitation research. There is no “gold standard” measure for “patient benefit” or consensus on which outcome measure(s) are optimal, and many studies use multiple measures to tap into different domains (Granberg et al. 2014). However, even for a specific concept such as hearing aid use, systematic reviews have shown a lack of consistency and robustness in the way hearing aid use is assessed and categorized (Perez & Edmonds 2012; Barker et al. 2014). Outcome measures to assess patient benefit need to be sensitive and appropriate for the intended method of benefit, and must not be too easy nor too difficult (Ferguson et al. 2014). It may be that an overall measure of hearing aid use (whether subjective or objective) is simply not sensitive nor appropriate to show the benefits of RLOs.

The long-term vision is to have educational resources, such as these RLOs, available to all hearing aid users in the UK. To this end, we have partnered with industrial and third sector partners and the RLOs, now branded as “C2Hear,” have been available to the UK audiology centers (NHS and independent sector) since end of 2014. An online version (C2Hear Online) is now freely available on YouTube. Further research needs to done to identify real-world benefits in a late phase clinical trial.

### Limitations

There were a number of limitations in this research. First of all, there was no active control group, so the participants could not be blinded with respect to the intervention. Similarly, although a number of precautions were taken to blind the research audiologists to the intervention and control groups (e.g., clinical audiologists recruited and randomized the patients and the research audiologists performed the evaluations), the explicit request for participants not to reveal their group was not always successful. A double-blind trial with an active control group (e.g., multimedia activities not associated with hearing) should be considered in future studies of this type. There was no long-term follow-up, which has been identified as major problem in hearing rehabilitation research (Barker et al. 2014). This was due primarily to limitations of time and funds imposed by the grant funding stream. However, in view of results from other patient education studies, which showed improvements in outcomes that were not evident shortly after the intervention but were revealed some months later (Kramer et al. 2005; Thorén et al. 2014), any future studies of this nature should include a long-term follow-up of at least 6 months. Despite a prospective study design, the study sample was not representative of the typical clinic population. Finally, cognitive abilities, which were not tested, could have helped inform whether cognition was a factor in the results.

### Future Research

With the increasing functionality available of online and smartphone technology, there are a number of potential future developments. Individualized tailoring of resources would provide a more user-centered intervention to meet individuals’ needs rather than the current “one-size-fits-all” approach taken in this research. This would allow the more technologically able to explore new technologies and reduce the basics, whereas for the less able, the converse would be more relevant. New technological developments in multilingual mobile translation (Albrecht et al. 2013) could also address those who have poor understanding of English, around 6% in this study. Indeed for this disadvantaged group, RLOs specific to their native language would be a very helpful facilitator in the delivery of hearing healthcare information. There was a clear involvement of communication partners, and focus groups were highly in favor of RLOs specifically targeted for communication partners. Development of these is currently underway. A pilot study of the RLOs used by care staff in residential care homes showed highly significant improvements in knowledge of hearing aids and communication (Rocks & Ferguson 2013). As around 10% of patients were unable to participate due to cognitive decline, the use of the RLOs could extend to caregivers, including family members as well as professional care workers. Other healthcare professionals, such as family doctors, could also benefit from tailored RLOs to highlight the need for early onward referral to Audiology, as only around 50% of 55- to 74-year-olds with significant hearing loss are referred (Davis et al. 2007). Finally, there is the opportunity for RLOs to help increase public awareness more generally to address the huge public health issues around hearing loss.

## CONCLUSIONS

This study developed an educational program for first-time hearing aid users based on the concept of RLOs. This concept has been trialed for the first time in audiology in one of the largest RCTs of an educational intervention for individualized use in adult auditory rehabilitation to date. The results of this interactive, multimedia educational intervention showed a range of benefits, suggesting that it may be a valuable supplement to the clinical management of first-time hearing aid users.

## ACKNOWLEDGMENTS

The authors thank Holly Thomas for collecting the data and Victoria Owen and Matthew Jones for analyzing some of the data. Special thanks to the media developers James Henderson and Michael Taylor, the public patient involvement panel (Anne Darby, Tina Wales, Rachel Ravenlock, Patricia Barnes), and our willing video stars (Pat, Chris, Richard, David, Tina, Caroline, Clive, Jan, Jill, Cedric) for their valuable input into developing the RLOs. The authors gratefully acknowledge the audiologists at Nottingham Audiology Services who recruited the participants (Julie Brady, Alissa Baguley, Karenbir Bath, Jeff Davies, Karen Goodrum-Clarke, Leena Kapilla, Annie Jones, Joanne Rowe), and special thanks to Helen Bastow. The authors also thank Ashana Tittle and Sandra Smith for their help with the data and Martin Morrison for setting up the internet portal. To view C2Hear Online RLOs, please go to the NIHR Nottingham Hearing Biomedical Research Unit website http://www.hearing.nihr.ac.uk/, or view the C2Hear Online RLOs via YouTube.

## References

[R1] Action on Hearing Loss (2011). Hear Me Out: Audiology Services in Scotland - Services Provided, Patients’ Experience and Needs.

[R2] Ainsworth S., Loizou A. (2003). The effects of self-explaining when learning with text or diagrams.. Cog Sci.

[R3] Albrecht U. V., Behrends M., Schmeer R. (2013). Usage of multilingual mobile translation applications in clinical settings.. JMIR Mhealth Uhealth.

[R4] Armero O. E. (2001). Effects of denied hearing loss on the significant other.. Hear J.

[R5] Barker F., Mackenzie E., Elliott L. (2014). Interventions to improve hearing aid use in adult auditory rehabilitation.. Cochrane Library.

[R6] Benedek J., Milner T. (2002). Measuring desirability: New methods for evaluating desirability in a usability lab setting.. Proceedings of the Usability Professional Association Conference.

[R7] Bertoli S., Staehelin K., Zemp E. (2009). Survey on hearing aid use and satisfaction in Switzerland and their determinants.. Int J Audiol.

[R8] Beynon G. J., Thornton F. L., Poole C. (1997). A randomized, controlled trial of the efficacy of a communication course for first time hearing aid users.. Br J Audiol.

[R9] Biggs J. B., Tang C. (2003). Teaching for Quality Learning at University, Society for Research into Higher Education and Open University Press.

[R10] Block M. (2001). Hearing Aid Repair Rates.

[R11] Boothroyd A. (2007). Adult aural rehabilitation: What is it and does it work?. Trends Amplif.

[R12] Brandreth M., Leighton P., Wharrad H. (2013). Development of interactive video tutorials to educate first-time hearing aid users: A participatory approach.. Int J Audiol.

[R13] Braun V., Clarke V. (2006). Using thematic analysis in psychology.. Qual Res Psychol.

[R14] British Society of Audiology (2008). Guidance on the use of real ear measurement to verify the fitting of digital signal processing hearing aids.. www.thebsa.org.uk/wp-content/uploads/2014/04/REM.pdf.

[R15] British Society of Audiology (2011). Pure-tone air- and bone-conduction threshold audiometry with and without masking.. www.thebsa.org.uk/wp-content/uploads/2014/04/BSA_RP_PTA_FINAL_24Sept11_MinorAmend06Feb12.pdf.

[R16] Brooke R. E., Isherwood S., Herbert N. C. (2012). Hearing aid instruction booklets: Employing usability testing to determine effectiveness.. Am J Audiol.

[R17] Caposecco A., Hickson L., Meyer C. (2014). Hearing aid user guides: Suitability for older adults.. Int J Audiol.

[R18] CETL (2009). http://www.rlo-cetl.ac.uk/whatwedo/evaluation/toolkit.php.

[R19] Chisolm T. H., Abrams H. B., McArdle R. (2004). Short- and long-term outcomes of adult audiological rehabilitation.. Ear Hear.

[R20] Chisolm T. H., Johnson C. E., Danhauer J. L. (2007). A systematic review of health-related quality of life and hearing aids: Final report of the American Academy of Audiology Task Force On the Health-Related Quality of Life Benefits of Amplification in Adults.. J Am Acad Audiol.

[R21] Cox R. M., Alexander G. C. (1999). Measuring satisfaction with amplification in daily life: The SADL scale.. Ear Hear.

[R22] Cox R. M., Alexander G. C. (2002). The international outcome inventory for hearing aids (IOI-HA): Psychometric properties of the English version.. Int J Audiol.

[R23] Davis A., Smith P., Ferguson M. (2007). Acceptability, benefit and costs of early screening for hearing disability: A study of potential screening tests and models.. Health Technol Assess.

[R24] Deloitte (2014). http://www2.deloitte.com/content/dam/Deloitte/global/Documents/Technology-Media-Telecommunications/gx-tmt-2014prediction-smartphone.pdf.

[R25] Desjardins J. L., Doherty K. A. (2009). Do experienced hearing aid users know how to use their hearing AIDS correctly?. Am J Audiol.

[R26] Dolan P. (1997). Modeling valuations for EuroQol health states.. Med Care.

[R27] Edelson P. J., Pittman V. V. (2001). http://www.sunysb.edu/spd/dean_papers/newdelhi.pdf.

[R28] El-Molla F., Smith Z., Henshaw H. (2012). Retention of rehabilitation information by first-time hearing aid users with and without interactive patient information.. British Academy of Audiology Conference Proceedings, Manchester.

[R29] Euroqual (1990). EuroQol-a new facility for the measurement of health-related quality of life.. Health Pol.

[R30] Ferguson M. (2014). Tackling information overload and retention – interactive multimedia videos for first-time hearing aid users.. ENT Audiol News.

[R31] Ferguson M. A., Henshaw H., Clark D. P. (2014). Benefits of phoneme discrimination training in a randomized controlled trial of 50- to 74-year-olds with mild hearing loss.. Ear Hear.

[R32] Ferguson M., Brandreth M., Brassington W. (2015). Information retention and overload in first-time hearing aid users: An interactive multimedia educational solution.. Am J Audiol.

[R84] Ferguson M. A., Leighton P. E., Brandreth M. (2012). Development of evidence-based interactive videos for first-time hearing aid users.. Int J Audiol.

[R33] Friedman L. M., Furberg C., DeMets D. L. (2010). Fundamentals of clinical trials.

[R34] Friemel T. N. (2014). The digital divide has grown old: Determinants of a digital divide among seniors.. New Media Soc.

[R35] Gatehouse S. (1999). Glasgow hearing aid benefit profile: Derivation and validation of client-centred outcome measures for hearing aid services.. J Am Acad Audiol.

[R36] Goggins S., Day J. (2009). Pilot study: Efficacy of recalling adult hearing-aid users for reassessment after three years within a publicly-funded audiology service.. Int J Audiol.

[R37] Granberg S., Dahlström J., Möller C. (2014). The ICF core sets for hearing loss–researcher perspective. Part I: Systematic review of outcome measures identified in audiological research.. Int J Audiol.

[R38] Greengross S. (2014). Commission on Hearing Loss: Final Report. In International longevity centre-UK (Ed.).

[R39] Henshaw H., Clark D., Kang S. (2012). Computer skills and internet use in adults aged 50–74 years: Influence of hearing difficulties.. J Med Internet Res.

[R40] Hibbard J. H., Mahoney E. R., Stockard J. (2005). Development and testing of a short form of the patient activation measure.. Health Serv Res.

[R41] Hickson L., Worrall L., Scarinci N. (2007). A randomized controlled trial evaluating the active communication education program for older people with hearing impairment.. Ear Hear.

[R42] Jacobson N. S., Follette W. C., Revenstorf D. (1984). Psychotherapy outcome research: Methods for reporting variability and evaluating clinical significance.. Beh Ther.

[R43] Kessels R. P. (2003). Patients’ memory for medical information.. J R Soc Med.

[R44] Kochkin S. (2000). Marke Trak V: “Why my hearing aids are in the drawer”: The consumers’ perspective.. Hear J.

[R45] Kochkin S. (2009). MarkeTrak VIII: 25-year trends in the hearing health market.. Hear Rev.

[R46] Koper E. J. R. (2003). Combining Re-usable Learning Resources and Services to Pedagogical Purposeful Units of Learning.

[R47] Kramer S. E., Allessie G. H., Dondorp A. W. (2005). A home education program for older adults with hearing impairment and their significant others: A randomized trial evaluating short- and long-term effects.. Int J Audiol.

[R48] Lin F. R., Metter E. J., O’Brien R. J. (2011). Hearing loss and incident dementia.. Arch Neurol.

[R49] Linssen A. M., Joore M. A., Theunissen E. J. (2013). The effects and costs of a hearing screening and rehabilitation program in residential care homes for the elderly in the Netherlands1.. Am J Audiol.

[R50] Lowe C. (2015). Under Pressure: NHS Audiology Across the UK.

[R51] Lundberg M., Andersson G., Lunner T. (2011). A randomized, controlled trial of the short-term effects of complementing an educational program for hearing aid users with telephone consultations.. J Am Acad Audiol.

[R52] McCormack A., Fortnum H. (2013). Why do people fitted with hearing aids not wear them?. Int J Audiol.

[R54] Medical Research Council (2008). Developing and evaluating complex interventions: new guidance.. http://www.mrc.ac.uk/documents/pdf/complex-interventions-guidance/.

[R85] Meyer C., Hickson L., Khan A. (2014). What is important for hearing aid satisfaction? Application of the expectancy-disconfirmation model.. J Am Acad Audiol.

[R55] Mosen D. M., Schmittdiel J., Hibbard J. (2007). Is patient activation associated with outcomes of care for adults with chronic conditions?. J Ambulatory Care Management.

[R56] Murray E., Davis H., Tai S. (2001). A randomised controlled trial of an interactive multimedia decision aid on hormone replacement therapy in primary care.. Br Med J.

[R57] NHS Scotland (2009). Quality standards for adult hearing rehabilitation.. http://www.scotland.gov.uk/Resource/Doc/270517/0080557.pdf.

[R58] NICE (2013). Guide to the methods of technology appraisal 2013.. https://www.nice.org.uk/article/pmg9/chapter/Foreword.

[R59] O’Keefe M., O’Regan L., Cashman D. (2008). Supporting the development of communities of practice: Informal versus formal communities.. Association for Learning Technology conference, “Rethinking the Digital Divide.”.

[R60] Perez E., Edmonds B. A. (2012). A systematic review of studies measuring and reporting hearing aid usage in older adults since 1999: A descriptive summary of measurement tools.. PLoS One.

[R61] Reese J. L., Hnath-Chisolm T. (2005). Recognition of hearing aid orientation content by first-time users.. Am J Audiol.

[R62] Rocks T., Ferguson M. (2013). Does training care-staff using interactive videos improve their hearing aid practical skills, understanding and perception of the importance of hearing aids?. British Academy of Audiology Conference Proceedings.

[R63] Scarinci N., Worrall L., Hickson L. (2008). The effect of hearing impairment in older people on the spouse.. Int J Audiol.

[R64] Scarinci N., Worrall L., Hickson L. (2012). Factors associated with third-party disability in spouses of older people with hearing impairment.. Ear Hear.

[R65] Schulz K. F., Altman D. G., Moher D. (2010). CONSORT 2010 Statement: Updated guidelines for reporting parallel group randomised trials.. Br Med J.

[R66] Sedgwick P. (2014). Clinical significance versus statistical significance.. Br Med J.

[R67] Statista (2015). http://www.statista.com/statistics/289180/household-penetration-of-dvd-players-in-the-uk/.

[R68] Stark P., Hickson L. (2004). Outcomes of hearing aid fitting for older people with hearing impairment and their significant others.. Int J Audiol.

[R69] Swanepoel D. W., Hall J. W. (2010). A systematic review of telehealth applications in audiology.. Telemed J E-Health.

[R70] Sweetow R., Sabes J. H. (2010). The communication confidence profile: A vital, but overlooked subjective domain.. Hear J.

[R71] Thorén E., Svensson M., Törnqvist A. (2011). Rehabilitative online education versus internet discussion group for hearing aid users: A randomized controlled trial.. J Am Acad Audiol.

[R72] Thorén E. S., Oberg M., Wänström G. (2013). Internet access and use in adults with hearing loss.. J Med Int Res.

[R73] Thorén E. S., Oberg M., Wänström G. (2014). A randomized controlled trial evaluating the effects of online rehabilitative intervention for adult hearing-aid users.. Int J Audiol.

[R74] UNECE (United Economic Commission for Europe) Statistical Database (2015). http://w3.unece.org/pxweb/dialog/Saveshow.asp?lang=1.

[R75] Ventry I. M., Weinstein B. E. (1982). The hearing handicap inventory for the elderly: A new tool.. Ear Hear.

[R76] Vuorialho A., Karinen P., Sorri M. (2006). Counselling of hearing aid users is highly cost-effective.. Eur Arch Otorhinolaryngol.

[R77] Whitmer W. M., Howell P., Akeroyd M. A. (2014). Proposed norms for the Glasgow hearing-aid benefit profile (Ghabp) questionnaire.. Int J Audiol.

[R78] Windle R. J., Wharrad H. (2010). Reusable learning objects in health care education interprofessional e-learning and collaborative work: Practices and technologies.

[R79] Windle R. J., McCormick D., Dandrea J. (2010). The characteristics of reusable learning objects that enhance learning: A case-study in health-science education.. Br J Educ Technol.

[R80] Wong L. L., Hickson L., McPherson B. (2003). Hearing aid satisfaction: What does research from the past 20 years say?. Trends Amplif.

[R81] Zhang J. (1997). The nature of external representations in problem solving.. Cog Sci.

[R82] Zhang D., Zhou L., Briggs R. O. (2006). Instructional video in e-learning: Assessing the impact of interactive video on learning effectiveness.. Inf Man.

[R83] Zigmond A. S., Snaith R. P. (1983). The hospital anxiety and depression scale.. Acta Psychiatr Scand.

